# Prediction model of quality of life using the decision tree model in older adult single-person households: a secondary data analysis

**DOI:** 10.3389/fpubh.2023.1224018

**Published:** 2023-08-31

**Authors:** Dajung Ryu, Sohyune Sok

**Affiliations:** ^1^Department of Nursing, Graduate School, Kyung Hee University, Seoul, Republic of Korea; ^2^College of Nursing Science, Kyung Hee University, Seoul, Republic of Korea

**Keywords:** aged, single-person households, health, quality of life, decision trees

## Abstract

**Background:**

Attention is drawn to the subjective health status and quality of life of older adult single-person households, whose number is gradually increasing as factors including low fertility, increased life expectancy, aging, and household miniaturization interact.

**Objective:**

The study was to identify predictors that affect the quality of life of single-person households aged 65 years or older and living in South Korea.

**Methods:**

A secondary data analysis design was used. Data included physical, mental, social, and demographic characteristics, subjective health status, and quality of life parameters of 1,029 older adult single-person households surveyed by the Korea Health Panel in 2019. For analysis, the predictive model was evaluated using split-sample validation and the ROC curve. The area under the curve after the decision tree analysis was calculated. Final nodes predicting the quality of life of older adult single-person households were derived.

**Results:**

Significant predictors were identified in this order: subjective health status, chronic disease, income, and age. Subjective health status was the most important factor influencing quality of life (△ *p* < 0.001, *x*^2^ = 151.774). The first combination that perceived high quality of life of older adult single-person households was the case of high subjective health status and no chronic disease, followed by the case of high subjective health status, presence of chronic disease, and high income.

**Conclusion:**

This study confirmed that subjective health status and chronic disease are essential factors for quality of life among the four related indicators of quality of life presented by the OECD. In nursing practice, nurses need to pay attention the factors influencing quality of life of older adult single-person households. Especially, nursing practice for older adult single-person households needs to be focused on improving subjective health status and on relieving chronic disease.

## Introduction

Life expectancy is increasing worldwide as the mortality rate improves owing to the expansion in the supply of medical resources, alleviation of problems in medical access, improvement in income level, and increased interest in improving quality of life. South Korea’s life expectancy in 2020 is 82.8 years. The rate of increase from the previous year is the highest at 97.4% among OECD member countries, and the proportion of the older adults is expected to increase further in the future (1). South Korea entered an aged society in 2017, and the expected proportion of the older adult population in 2050 is 35.9%, which is expected to rank second among countries in the world (1–4). With the spread of individualistic ideology and the diversification of individual values, fertility rate is expected to continue to decrease, and the aging population will intensify. Moreover, the recent notable demographic change in South Korea involves the downsizing of households. The number of Korean single-person households aged 65 years or older increased by 36% from 1.06 million in 2010 to 1.44 million in 2018, accounting for 7.2% of the total number of households (1, 3). In the future, the number of older adult single-person households is expected to continue to increase due to the aging population and the deepening of trends in terms of low fertility, individual economic and health independence, enjoyment of personal life, and changes in parental support preferences (3, 5).

In South Korea, older adult single-person households have a high proportion of no economic activity, low asset sufficiency, and stability as well as a large gap in health (4, 6). Compared to multi-person households, they lack psychological and social stability, having poor health status, and poor access to medical care (7, 8). Emotional loneliness in older adults single-person households is associated with mortality, higher rates of depression, and lower quality of life (9). In other words, older adult single-person households comprise a group that needs special attention, and they need help when they have a disease or disability that require long-term care services. As this puts a burden on individual health and social welfare, it is necessary to study the health status and health outcomes of older adult single-person households. Exploring the health determinants of older adult single-person households is an essential task to improve the quality of life. It is also important to set and evaluate goals for the effectiveness and equity of health-related policies.

The health of the older adults refers to a state of complete physical, mental, and social well-being while maintaining autonomy, independence, and intergenerational solidarity (3, 10). Four indices of life expectancy, avoidable death, chronic disease, and subjective health status of the older adults are key factors for quality of life (4, 11). Understanding subjective health status has become essential to improving quality of life. Subjective health status refers to the overall evaluation or perception of an individual’s health and is a good factor in evaluating health and quality of life (11, 12). Regardless of the diagnosis of a medical institution, the physical and mental health status felt by an individual is a predictor of objective indicators such as mortality rate (13, 14), even though it is a subjective characteristic, and comprehensively reflects the health status. Additionally, older people think their health is worse than younger people, which partially reflects the burden of chronic diseases (11). Chronic disease, which is an objective indicator, is a clinical diagnosis characterized by vulnerability and is known as an unfavorable predictor of health and quality of life. However, even in the case of taking medications for chronic diseases, the person can maintain a smooth daily life and think that his/her health is good. Even when one is not taking special medications, the person can underestimate his/her health status by worrying and being excessively concerned about his/her health.

Quality of life is another important concept to be considered in relation to subjective health status. Quality of life is multidimensional according to the content and measurement method (15), and it is abstract as it contains individual values and philosophies. The WHO defines quality of life as an individual’s perception for one’s place within the culture and value system related to the subject’s goals, expectations and standards (10). Looking at the previous studies related to quality of life, there were many comparative studies between tools for measuring quality of life (16, 17). Most of the studies related to influencing factors were based on chronic diseases (16–18), and studies related to subjective health status were limited to some age groups or gender (19–22). There is lack of research on which predictor, either chronic disease or subjective health status, is a better predictor as an influencing factor on the quality of life of older adult single-person households (11).

Decision tree analysis is a method of classifying and predicting vast amounts of data. It is a useful tool in the health care field because it is easier to understand and facilitates prediction of characteristics by visualizing the predicted rule as a tree (23–25). Quality of life in the older adults is related to physical (26, 27), psychological (28), and socioeconomic (29, 30) factors. Therefore, we aimed to more intuitively and objectively identify the predictors of quality of life for older adults and single-person households through various combinations of variables related to quality of life. Decision tree analysis models that present a holistic picture of predictors of quality of life can aid in better clinical decision-making than individual predictors in regression models (23). Accordingly, this study intends to contribute to facilitating the development of community-based programs for improvement of quality of life by examining whether physical, mental, social, and demographic characteristics appear as significant influencing factors in older adult single-person households according to subjective health status. Focus is on the four OECD indicators that are related to or suggested as influencing factors.

The purpose of this study sought to determine the path for predicting the quality of life of older adult single-person households in South Korea using the decision tree analysis method. The specific aims were (1) to identify factors related to the quality of life of older adult single-person households; (2) to identify the good and poor quality of life groups in the older adult single-person households.

## Methods

### Study design

This study was a secondary data analysis study designed to build a predictive factor model that affects the quality of life of older adult single-person households using the Korea Health Panel 2019 Annual Data Version 2.0.1 (31) hosted by the Korea Institute for Health and Social Affairs and the National Health Insurance Service.

### Study subjects and data collection

The Korea Health Panel Survey is a nationally approved statistic (Approval No. 920012). Sampling secured representativeness through the standard sample design of the 2016 Population and Housing Census in Korea, which reflects changes in the demographic structure of the population, such as aging. Based on the data of the Population and Housing Census, it was conducted by a probability-proportional two-step stratified colony sampling method in which administrative districts were set as stratification variables, and this data is the most up-to-date data as the second-term panel data. The weight of the Korea Medical Panel data can statistically correct the bias of the data, and the representativeness and accuracy of the estimate can be improved by correcting the inclusion error, unequal sampling rate, and non-response error due to the difference in the number of households and population between the time of sample design and the time of survey. Among 1,440 single-person households out of a total of 6,689 households that responded to the 2019 Korea Health Panel Survey, 1,029 older adult people aged 65 years or older responded to all the survey variables in the study. This study was conducted from April to October 2022.

### Measurements

This study included the physical, mental, and socioeconomic factors presented in previous studies, focusing on the four indicators (11) of the OECD, which were presented as factors influencing quality of life, to identify the path to predict the quality of life of single-person older adult households (26–30).

### General and health-related characteristics list

General and health-related characteristics list included age, gender, income, educational level, chronic disease, disability, stress, suicide thoughts, need care, commercial treatment center, and self-rated health (SHR). This consisted of a total of 11 items.

### Physical factors

For physical factors, chronic disease and presence of disability were used. In this study, only those diagnosed by a doctor were included for chronic disease, resulting in 28 types other than hypertension and diabetes (31). These include major chronic diseases affecting the older adults as suggested by the WHO and avoidable causes of death as suggested by the OECD: cardiovascular disease, hypertension, cerebrovascular disease, diabetes, cancer, respiratory disease, and musculoskeletal disease (10, 11). The question about disability was “Do you currently have a disability?’

### Mental factors

For mental factors, the stress level and the presence of suicidal thoughts were used. Stress consisted of a 4-point Likert scale (1 = very much, 4 = hardly). In this study, the score was reverse-coded: the higher the score, the more severe the stress. The question about suicidal thoughts was on a nominal scale and “Have you ever had thoughts of wanting to die in the past 1 year?”

### Social factors

For social factors, the need for care and the presence of the usual source of care were used to confirm the level of social support of the subject. The need for care was divided into cases in which social care was required due to physical and mental health problems and cases in which social care was not required. The presence of the usual source of care was a factor related to the existence of physical and human resources in the local community. The question about the presence of the usual source of care was whether the person had a medical institution and a doctor that he/she usually visited. When the person had both, it was determined that the person had the usual source of care.

### Subjective health status

Subjective health status answered in the form of self-assessment was a comprehensive evaluation of the subject’s overall health status. The question for this was ‘How is your current health?’ It consisted of a 5-point Likert scale (1 = very good, 5 = very bad). In this study, the score was reverse-coded: the higher the score, the better the subjective health status.

### Quality of life

The quality of life scale used was the EQ-5D-3L developed by the EuroQoL group, consisting of the dimensions of exercise ability, self-management, activity of daily living, pain and discomfort, and anxiety and depression. Each dimension consisted of 3 levels (1 = no disturbance, 2 = somewhat disturbing, 3 = very disturbing). Therefore, a total of 3^5^ = 243 combinations was made to measure the quality of life according to different health status levels. A case in which all five dimensions were level 1 was regarded as a state of complete health, and the value at this time was set to “1”. If the dimension was answered as level 2 or 3, the value was calculated using the weight formula. In this study, the quality-weighted correction score was used to calculate with the method suggested by the Korea Centers for Disease Control and Prevention (32). The range of the subject’s quality of life score was −0.171 to 1, and the codes and calculation formulas for dimensions and levels are shown in Table 1.

**Table 1:** EQ-5D-3L index (*N* = 1,029).

	Level 1	Level 2	Level 3
Exercise ability		M2	M3
Self-management		SC2	SC3
ADL		UA2	UA3
Pain, discomfort		PD2	PD3
Anxiety, depression		AD2	AD3
Interaction			N3
EQ-5D index =1-(0.05 + 0.096 × M2 + 0.418 × M3 + 0.046 × SC2 + 0.136 × SC3 + 0.051 × UA2 + 0.208 × UA3 + 0.037 × PD2 + 0.151 × PD3 + 0.043 × AD2 + 0.158 × AD3 + 0.05 × N3)			

ADL, Activities of Daily Living.

### Ethical considerations

Statistical data of the Korea Health Panel were obtained with prior consent from all subjects before data collection. Data were analyzed after the data access review was approved by submitting the consent form for data use before the start of the study. The study was conducted according to the guidelines of the Declaration of Helsinki, and approved by the Institutional Review Board of the Korea Institute for Health and Social Affairs (Approval No. 2022-017 and April 15, 2022).

### Data analysis

The data were analyzed using the SPSS 25.0 program. Descriptive statistics were performed on the subjects’ characteristics and quality of life, and independent samples t-test and *x*^2^-test were carried out by dividing the subjects into two groups based on the mean quality of life calculated by the quality-weighted correction score to compare the subjects’ characteristics. In order to predict factors related to the quality of life of single-person older adult households, a decision tree analysis method among data mining techniques was applied. Decision trees are composed of components called nodes, and the separation process of nodes occurs according to the frequency belonging to each category of the target variable (33). In this study, the *x*^2^-test was used when the target variable was discrete, and the Chi-squared Automatic Interaction Detection (CHAID) algorithm was used to allow separation of two or more using F-test when the target variable was continuous (34). That is, the separation criterion for forming the structure of the decision tree is the chi-square test statistic, the significance level of the node split is set to.05, and the maximum number of iterations for model estimation is set to 100. The stopping rules of a decision tree refer to rules that make the current node become a terminal node, and these rules require the maximum tree depth and the minimum number of observations in a node (24). In this study, the maximum number of levels of CHAID was specified as 3, Parent node 100, and Child node 50, and a split sample validity test, ROC curve, and area under the curve were performed to examine whether the decision tree could be applied to the population.

## Results

### General and health-related characteristics of the study participants

The mean age of the subjects was 76.48 ± 6.79 years, and there were more females (78.3%) than males. Most of the subjects (88.3%) had chronic diseases. In terms of stress, 41.6% hardly felt stress, 39.8% felt a little, 16.1% felt a lot, and 2.5% felt very much. 88.0% of the subjects answered that they had no suicidal thoughts, and 93.0% of the subjects answered that they did not need care. 55.0% of subjects had the usual source of care. The mean score of subjective health status was 2.91 ± 0.89 points (range 1 ~ 5), and 33.5% was perceived as bad. Examination of the difference in the general characteristics according to the quality of life after dividing the subjects into two groups based on the mean quality of life of 0.862 ± 0.14 showed that all variables differed between the two groups (Table 2).

**Table 2:** General and health-related characteristics of the study participants (*N* = 1,029).

Categories	Total	Low QoL (*n* = 456)	High QoL (*n* = 573)	*χ* ^2^ or *t* (*p*)	Cramer’s V
	*n* (%) or Mean ± SD	*n* (%) or Mean ± SD	*n* (%) or Mean ± SD		
Age (year)	76.48 ± 6.79	78.40 ± 6.75	74.95 ± 6.43	8.384 (< 0.001)	
Gender					
Male	223 (21.7)	69 (15.1)	154 (26.9)	20.633 (< 0.001)	0.133
Female	806 (78.3)	387 (84.9)	419 (73.1)		
Income (10,000KRW/year)	1372.99 ± 1812.75	1077.11 ± 688.84	1608.46 ± 2324.42	−5.193 (< 0.001)	
Educational level					
High school below	789 (76.7)	393 (86.2)	396 (69.1)	42.278 (< 0.001)	0.200
High school	176 (17.1)	43 (9.4)	133 (23.2)		
College above	64 (6.2)	20 (4.4)	44 (7.7)		
Chronic disease					
Yes	909 (88.3)	442 (96.9)	467 (81.5)	58.676 (< 0.001)	0.240
No	120 (11.7)	14 (3.1)	106 (18.5)		
Disability					
Yes	137 (13.3)	92 (20.2)	45 (7.9)	33.405 (< 0.001)	0.186
No	892 (86.7)	364 (79.8)	528 (92.1)		
Stress					
Barely felt	427 (41.6)	163 (35.7)	264 (46.1)	25.996 (< 0.001)	0.217
Feel a little	410 (39.8)	178 (39.1)	232 (40.5)		
Feel a lot	166 (16.1)	101 (22.1)	65 (11.3)		
Feel very much	26 (2.5)	14 (3.1)	12 (2.1)		
Suicide thoughts					
Yes	123 (12.0)	76 (16.7)	47 (8.2)	17.285 (< 0.001)	0.144
No	906 (88.0)	380 (83.3)	526 (91.8)		
Need care					
Yes	72 (7.0)	65 (14.3)	7 (1.2)	66.277 (< 0.001)	0.252
No	957 (93.0)	391 (85.7)	566 (98.8)		
Commercial treatment center					
Yes	566 (55.0)	263 (57.7)	303 (52.9)	7.312 (0.026)	0.091
No	463 (45.0)	193 (42.3)	270 (47.1)		
SRH	2.91 ± 0.89	2.44 ± 0.80	3.29 ± 0.76	−17.320 (< 0.001)	0.529
Very bad	44 (4.3)	42 (9.2)	2 (0.3)		
Bad	300 (29.2)	218 (47.9)	82 (14.3)		
Moderate	407 (39.5)	151 (33.1)	256 (44.7)		
Good	258 (25.1)	43 (9.4)	215 (37.6)		
Very good	20 (1.9)	2 (0.4)	18 (3.1)		

QoL, Quality of Life; SRH, Self-Rated Health; SD, Standard Deviation.

Bold values denote statistical significance at the *p* < 0.05 level.

### Subjective health status and level of quality of life and relationship

The quality of life dimension of subjects was measured using a weight formula. The closer to 1 the value obtained is, the more positively they perceived their life. The mean quality of life of the subjects was 0.862 ± 0.14. The mean of the self-management dimension was the highest at 0.867 ± 0.33, and the mean of the pain and discomfort dimension was the lowest at 0.416 ± 0.47. The subjects’ quality of life had a moderate correlation with subjective health status (*r* = 0.55, *p* < 0.001), and it was confirmed that there was a significant correlation in the order of exercise ability (*r* = 0.47, *p* < 0.001), activity of daily living (*r* = 0.47, *p* < 0.001), pain and discomfort (*r* = 0.43, *p* < 0.001), self-management (*r* = 0.34, *p* < 0.001), and anxiety and depression (r = 0.33, *p* < 0.001) (Table 3).

**Table 3:** Means of and correlations among variables (*N* = 1,029).

Variables	Application of weighted value			Relationships with SRH
	Min	Max	Mean ± SD	*r* (*p*)
Quality of life	−0.056	1	0.862 ± 0.14	0.55 (< 0.001)
Exercise ability	0.096	1	0.614 ± 0.45	0.47 (< 0.001)
Self-management	0.046	1	0.867 ± 0.33	0.34 (< 0.001)
ADL	0.051	1	0.702 ± 0.44	0.47 (< 0.001)
Pain and discomfort	0.037	1	0.416 ± 0.47	0.43 (< 0.001)
Anxiety and depression	0.043	1	0.852 ± 0.35	0.33 (< 0.001)

SRH, Self-Rated Health; ADL, Activities of Daily Living; SD, Standard Deviation.

Bold values denote statistical significance at the *p* < 0.05 level.

### Prediction model of factors influencing the quality of life of subjects

**Figure 1:**
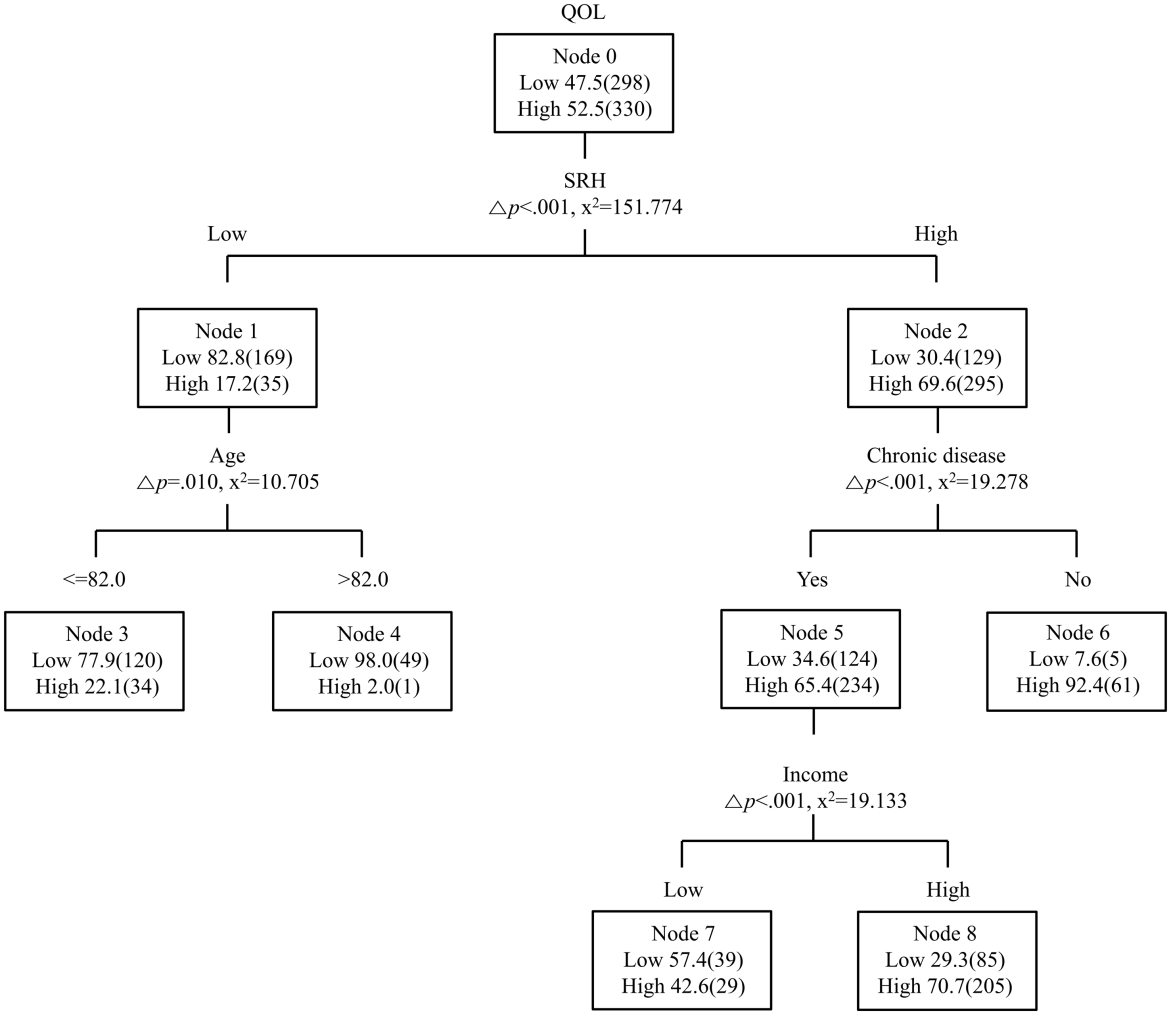
Decision tree for quality of life. QOL, Quality of Life; SRH, Self-rated health; Δ, adjusted; (), test data.

In order to build a model for predicting the quality of life of subjects, decision tree analysis was performed on the training data and test data by inputting all the variables used in the study. The variable of the parent node means the more important it is as an influencing factor, and the level of quality of life changes as the characteristics of the child node accumulate. In this study, the total number of nodes was 9, the final number of nodes was 5, and the number of levels was 3. The significant predictors were subjective health status, chronic disease, income, and age in this order. According to the decision tree analysis results, the most important factor influencing quality of life was subjective health status (△*p* < 0.001, *x*^2^ = 151.774). There were 5 combinations of cases where the quality of life was perceived as high, and the combination of the highest factors was the case of high subjective health status and no chronic disease (92.4%). The combination of second highest factors was the case of high subjective health status, presence of chronic disease, and high income (70.7%; Figure 1).

### Evaluations of predictive model

**Figure 2:**
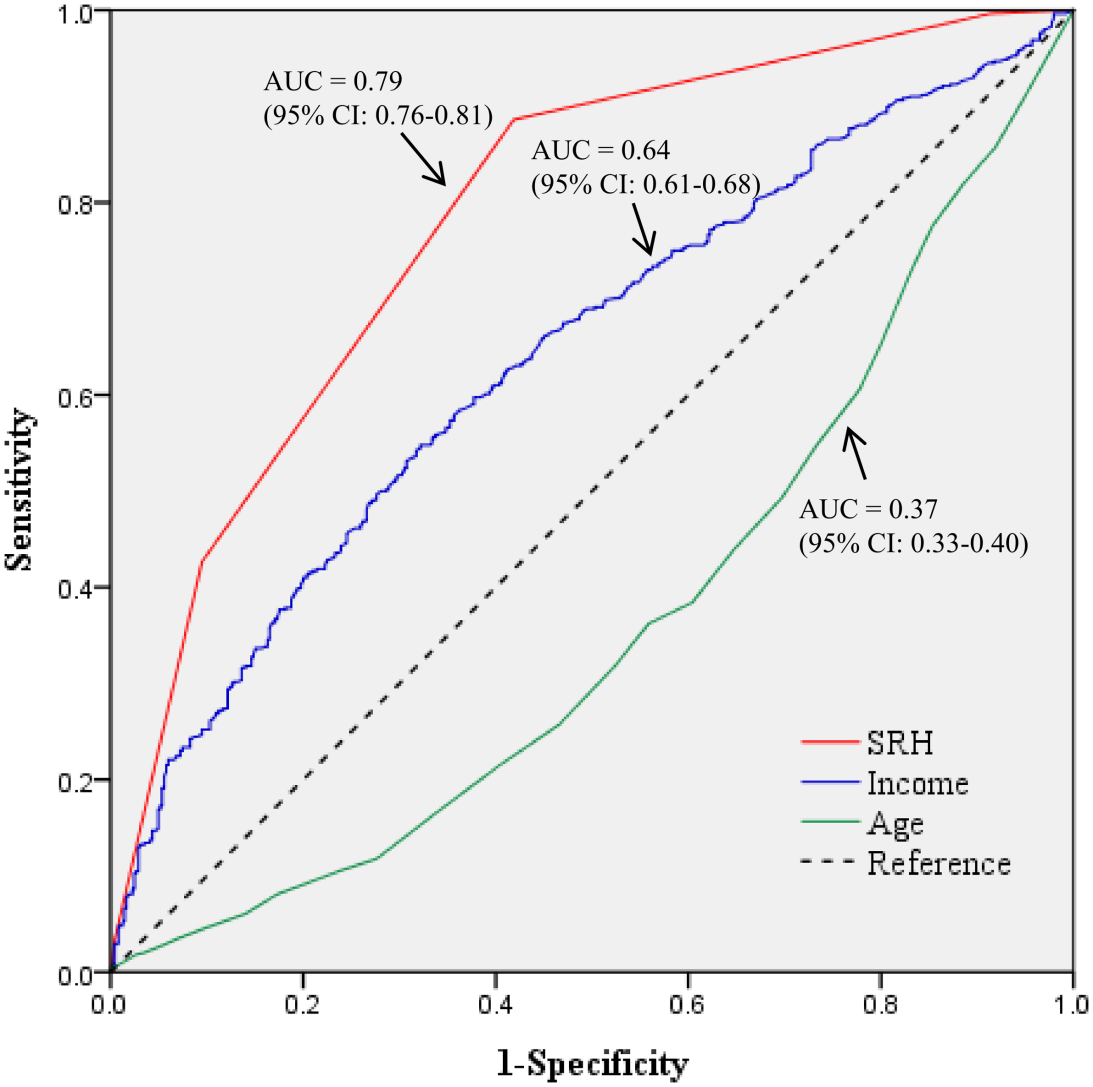
The receiver operating characteristic curves of predicting model. AUC, Area Under the Curve; CI, Confidence Interval.

In order to secure the suitability of the decision tree model, split-sample validation was performed by dividing the ratio of training data and test data to 5:5. The same structure as shown in Figure 1 was confirmed to ensure the validity of the model, and in the case of the training data, the risk estimate was 0.24, confirming that the probability of correct classification was 75.5%. In this study, the AUC value of subjective health status was 0.79 (95% CI = 0.76–0.81), the AUC value of income was 0.64 (95% CI = 0.61–0.68), and the AUC value of age was 0.37 (95% CI = 0.33–0.40), which was similar to the prediction ranking of the decision tree model (Table 4; Figure 2).

**Table 4:** Risk chart of decision trees.

Variables	RE	SE	%
Training data	0.24	0.02	75.5
Test data	0.29	0.02	70.8

RE, Risk Estimate; SE, Standard Error.

## Discussion

In an aging society that is intensifying along with economic development, the health status and quality of life of the older adults are attracting considerable attention. Quality of life reflects physical and mental health, which has been widely verified using standardized tools as having a significant correlation with health status (35, 36). Older adults’ health is closely related to access to economic and medical resources (37), and single-person households lacking family care are vulnerable in terms of health and emotional cognition (38–40). Therefore, in this study, in order to identify factors affecting the quality of life of older adult single-person households in South Korea, decision tree analysis using data mining technique was performed to model 5 predictive pathways.

In this study, the subjective health status, chronic disease, income, and age of older adult single-person households were found to affect the quality of life, supporting the results of previous studies (4, 16–18, 20, 21, 35, 41). First, subjective health status was identified as the most influential factor on the quality of life of older adult single-person households. Subjective health status can better represent a subject’s comprehensive health status and quality of life even if objective health status is considered (42–44). Based on the results, the subjective health status of the group with low quality of life was lower than the average, and that of the group with high quality of life was higher than the average. In other words, subjective health status evaluation can be an excellent method of selecting subjects for quality of life improvement since the vulnerable group and the high-risk group are identified. Therefore, accurate evaluation of subjective health status is important, and two methods are proposed. First, subjective health status is not based only on objective health indicators, but also on the complex action of physiological, psychological, social, and cultural factors (45). It is necessary to understand various concepts of health and to recognize the cultural custom of expressing the evaluation of subjective health status as a positive or negative opinion (45). Second, in addition to the scale of subjective health status presented as an existing ranking scale, it is also to evaluate one’s own health status compared to those of the same age. In a study investigating the tendency of the subjective health status of the older adults in the United States, it was found that the subjective health status of middle-aged adults aged 45 to 54 years deteriorated over time (46). Similarly, a study examining changes in the subjective health status of Korean adult single-person households reported that their subjective health status deteriorated over time and faster than that of multi-person households (47). In summary, it can be seen that in order to improve the subjective health status of older adult single-person households, it is necessary to provide health information and education on functional status from middle-aged adulthood. Moreover, when comparing the subjective health status of older adult single-person households in South Korea with other countries, the low subjective health status rate of South Korea is similar to that of Taiwan (33.1%) and higher than that of Japan (26.9%) and Shanghai (15.2%) (48–50). This means that the subjective health status of older adult single-person households in South Korea is particularly poor among major Asian countries. Follow-up research and theoretical development are needed to improve the subjective health status for this particular population.

The second factor affecting quality of life was identified as chronic disease. The improvement of medical technology and the expansion of the supply of medical resources indicate that many people suffer from chronic diseases, which is an important topic in the health care field. Chronic disease causes deterioration of body functions, and is an important predictor of health deterioration, especially in the older adults (44, 51). When the number of chronic diseases is small or absent, health status assessment will be improved. For example, Alzheimer’s disease, the most common neurodegenerative disease in the older adults, begins with symptoms of cognitive decline (52). Evaluation of various biomarkers for early identification of mild cognitive impairment can reduce the morbidity of chronic diseases. The next factors that affect quality of life were income and age. Income is an essential resource for maintaining and promoting health, which can help the subject effectively cope with health problems and is related to the cost of diet and exercise to maintain health. It is in the same context as the results reported in a previous study that income is highly related to subjective health status and well-being (53). Aging affects quality of life due to deterioration of physical, mental, and social functions as well as an increase in chronic diseases. Based on the results of this study, it is worth noting that subjective health status and chronic disease are major influencing factors on quality of life, but the strongest influencing factor is subjective health status. Subjective health status is a complex judgment about the severity of chronic diseases, and is a higher-level concept that includes symptoms of chronic diseases and undiagnosed diseases (43). The severity of chronic diseases differs from individual to individual, and quality of life is an issue that cannot be explained only by existing diseases and symptoms. Subjective health status is a dynamic viewpoint that evaluates not only the current health level but also the future health level. Chronic diseases and age are irreversible, but subjective health status can be modified, and individual health behaviors can be promoted to improve the positive growth and quality of life of the subject. This suggests that subjective health status should be evaluated independently in future studies.

The first combination that perceived quality of life as high was the case of high subjective health status and no chronic disease, followed by the case of high subjective health status, presence of chronic disease, and high income. The first combination that perceived quality of life as low was the case of low subjective health status and age 82 years or older. These results show there is a need for a community-based customized care program that can classify older adult single-person households that include both characteristics of low quality of life as a priority for improving the quality of life and subjective health status. In addition, chronic disease prevention and management programs should be expanded further since chronic diseases are important for older adult single-person households with high subjective health status. This result identified various approaches to improving the quality of life of older adult single-person households, prioritized management targets considering the combination, and suggested that a specific approach strategy was needed.

### Implications for practice, policy, and research

Since the subjective health status of middle-aged adults confirmed in previous studies also affects old age, it is necessary to provide health information and education on functional status in middle-aged adulthood. A community-based customized care program is required for old age. In addition, chronic disease prevention and management programs should be expanded further as chronic diseases are critical for older adult single-person households with high subjective health status. Chronic disease-specific care can be provided in the primary health care system, such as self-management support, decision support, and delivery system Design (54). This can ultimately improve the quality of life of older adult single-person households.

Based on the results of this study, the following suggestions are made. First, in providing programs to improve the quality of life of the older adults, the older adult single-person household should be given primary importance because it is a vulnerable group. Second, although the EQ-5D-3L has good measurement properties, it has been shown to have a ceiling effect in some studies, so replication studies using various quality of life tools are needed. Third, decision tree analysis is an effective method to classify a large amount of data and predict both categorical and continuous values (24), but it is difficult to determine the effect of each predictor variable like logistic regression analysis. Therefore, it is required to compare the predictive power of models constructed by performing complex sample logistic regression analysis to confirm the change in explanatory power and influence of explanatory variables by sequentially introducing them.

### Limitations

This study has several limitations. It was not possible to use various tools to evaluate the quality of life as a secondary data analysis study. Also, it was not possible to evaluate the difference in the change in quality of life since only one-year data were used.

## Conclusion

This study examined the characteristics of older adult single-person households using data from the Korea Health Panel and presented an approach strategy in practice by constructing a predictive model for improving the quality of life using decision tree analysis. In conclusion, the path for predicting the quality of life of older adult single-person households differed according to the subjective health status. Our study makes a significant contribution to the literature because of the novelty and innovation of this work and the direct practical relevance of our findings to informing research directions and medical guidelines. This paper will be of interest to the readership of your journal because of our focus on a highly understudied topic of importance within the interdisciplinary field of public health.

## Data availability statement

Publicly available datasets were analyzed in this study. This data can be found here: the Korea Health Panel 2019 Annual Data Version 2.0.1 (25) hosted by the Korea Institute for Health and Social Affairs and the National Health Insurance Service.

## Ethics statement

All procedures performed in this study involving human participants were in accordance with the ethical standards of the institutional and/or national research committee and with the 1964 Helsinki declaration and its later amendments or comparable ethical standards. The study was approved by the Institutional Review Board of statistical data of the Korea Health Panel (Approval No. 2022-017).

## Author contributions

DR and SS: conceptualization, methodology, validation, formal analysis, data curation, writing-original draft preparation, and writing-review and editing. SS: supervision. All authors contributed to the article and approved the submitted version.

## Conflict of interest

The authors declare that the research was conducted in the absence of any commercial or financial relationships that could be construed as a potential conflict of interest.

## Publisher’s note

All claims expressed in this article are solely those of the authors and do not necessarily represent those of their affiliated organizations, or those of the publisher, the editors and the reviewers. Any product that may be evaluated in this article, or claim that may be made by its manufacturer, is not guaranteed or endorsed by the publisher.
